# Sectoral Analysis of Food Waste in EU Countries: Implications for Pro-Environmental Orientation and Policy

**DOI:** 10.3390/foods15060972

**Published:** 2026-03-10

**Authors:** Marcela Taušová, Katarína Čulková, Maksym Mykhei, Peter Tauš, Lucia Domaracká, Alexandra Vraštiaková

**Affiliations:** Faculty BERG, Technical University of Košice, Letná 9, 042 00 Košice, Slovakia; marcela.tausova@tuke.sk (M.T.); maksym.mykhei@tuke.sk (M.M.); peter.taus@tuke.sk (P.T.); lucia.domaracka@tuke.sk (L.D.); alex.vrastiakova@gmail.com (A.V.)

**Keywords:** waste management, food wastage, circular economy, environmental policy, EU countries

## Abstract

Food waste remains a critical sustainability challenge for the European Union (EU), with significant negative impacts on environmental resources, economic efficiency, and social equity. This paper presents a comprehensive analysis of food waste across EU member states during the 2020–2023 period, examining waste generation across five key sectors: households, food service (restaurants and catering), retail, food manufacturing, and primary agriculture. The study uses Eurostat statistical data, standardising measurements to kilograms per capita and absolute tonnage to enable cross-country comparisons. Particular attention is devoted to the impacts of the COVID-19 pandemic, which disproportionately affected the service and retail sectors. Beyond descriptive analysis, the research investigates potential relationships between major economic indicators (Gross Domestic Product [GDP], median income, and material deprivation) and food waste rates, employing Kruskal–Wallis statistical tests to examine sectoral and cross-national patterns. Contrary to conventional assumptions, analyses reveal no statistically significant direct correlation between economic prosperity and waste generation, suggesting that institutional design, infrastructure availability, consumer awareness, and education exert greater determinative influence than aggregate wealth. Results demonstrate that households are the largest source of food waste across the EU, accounting for approximately 50% of food waste. At the same time, sectoral variations reflect country-specific structural and regulatory factors rather than levels of economic development. The research concludes with actionable policy recommendations targeting three intervention levels: individual behaviour change (consumer education, digital tools, and purchase planning), community infrastructure (food redistribution networks and collective composting), and institutional reform (regulatory harmonisation, circular economy incentives, and extended producer responsibility). These recommendations align with EU strategic priorities, including the European Green Deal, Farm to Fork Strategy, and 2030 Circular Economy Action Plan, with the specific objective of halving food waste by 2030 to enhance both environmental sustainability and food security.

## 1. Introduction

Food waste represents a serious global problem with far-reaching environmental, economic, and social consequences. However, whilst this phenomenon is studied at the global level, a specific knowledge gap exists regarding sectoral differences in food waste levels across EU member states and the potential relationships between this waste and socio-economic factors in individual countries. Food waste not only has environmental consequences, including greenhouse gas emissions, pollution, and the waste of natural resources, but also poses economic and ethical problems. Therefore, this paper addresses the complex issue of food waste, which is perceived as a key challenge for sustainable development in the European Union.

This paper aims to provide a comprehensive overview of the current state and development of food waste, identify the main causes of this phenomenon across sectors, and propose recommendations to improve food management in the Slovak Republic and the EU more efficiently and sustainably. The findings can contribute to more effective policy setting and to shaping a more sustainable approach to food across society.

Unless urgent action is taken to reduce food waste, 66 tons of food will be wasted every second worldwide by 2030 [1]. The United Nations has proposed halving food wastage by 2030, but in the next period, it will increase in most regions of the world, especially in Asia. The prognosis is waste growth in industrialised countries with growing populations [1].

An international review of the literature found a shortage of data on food waste, and estimates reported discrepancies [2]. There is therefore a need to research the subject area further. Marston et al. reviewed the literature to evaluate the potential to reduce water and food waste [3].

Also, according to Jesmwani et al., significant knowledge gaps remain in the research area, particularly regarding environmental influences [4]. There is therefore a need to consider such influences in formulating strategies to reduce food waste. Such strategies are implemented in several countries; for example, France established political and other measures to force traders to redistribute food and use food surpluses as animal feed [5]. The results demonstrated that retailers donating high-value products also achieved lower costs and greater overall environmental savings. However, a regulatory tool at the government level can be undermined by poor administration of the autonomy [6]. Therefore, a “portfolio” of strategies should be included with all interested parties. This includes, for example, ecological reporting, which is considered in countries such as the USA, Australia, Canada, and the EU, as well as in China, Brazil, India, and Mexico, to promote ecological responsibility in food processing [7]. In Europe, France, the UK, Belgium, the Netherlands, and Switzerland are countries with the highest potential for innovation in reducing food waste. The absence of governmental regulation and an environmental framework could be an obstacle to engaging company management in efforts to achieve food sustainability [8]. Therefore, the EU presented several scenarios for 2030 and 2050 [9]. Until 2050, it is necessary to monitor the rapid growth in animal product consumption, particularly in developed countries [10].

Another important reason to conduct more research on food waste is that food and beverage production is the second most energy-intensive sector, thereby worsening energy efficiency [11]. It is linked to greenhouse gas emissions, and companies and consumers should work together to reduce emissions and food waste simultaneously [12]. This will help achieve sustainability of the agricultural and food systems [13]. Despite efforts to reduce emissions, the global food system is connected to other sources of emissions, which could hinder achieving this goal [14]. CO_2_ emissions vary by food type, and analyses are mostly conducted in agricultural countries, compared with industrial ones [15]. Therefore, the EU identified the marked improvement in food waste treatment as a key factor for achieving carbon neutrality, when prevention, reuse, recycling, and energy recovery are supported [16].

Economic factors and sustainability are represented by food waste indicators, including food prices, food trade, the Sustainable Development Goals index, and GDP per capita [17]. This means that higher GDP is associated with stronger sustainability performance, as reflected in lower food waste. According to Economou et al., infrastructure, available knowledge, and social conditions influence the development of food waste [18]. Food waste is also linked to the search for plastic packaging waste [19], since plastic plays an important role in food security and durability. To address food-related plastic waste, bioplastics offer an attractive alternative to conventional packaging [20]. However, Gencia and Balan (2024) found that, contrary to expectations, higher GDP is not consistently correlated with lower household food waste [21]. This finding underscores the need for deeper sectoral and multi-country analysis.

It is important to study the area by source of food waste, such as households and schools. The volume of food waste is rising in schools [22]. Households produce over 53% of food waste, which is closely linked to the information and behaviour of food users. These areas demand optimisation of the household and school waste economy systems [23].

The results of the literature review show that the analysed subject presents a broad, multifaceted problem that spans all phases of the food supply chain [24]. Multi-criteria analysis has proven to be the best approach to studying food waste, and addressing it could yield positive social and economic impacts.

This study aims to provide a comprehensive assessment of the current state and evolution of food waste in the European Union, identify the principal drivers of food waste generation, and offer policy-relevant recommendations to enhance food management efficiency and sustainability across the EU. Specifically, the authors analyse the magnitude and determinants of food waste across five stages of the food supply chain (households; food service; retail and distribution; primary production; and food and beverage manufacturing) with a focus on developments from 2020 to 2022, emphasising policy initiatives such as food banks, community fridges, and retailer-led programmes. The work also scrutinises the regulatory framework, environmental behaviour across countries, and practical measures and best practices, including redistribution mechanisms. It assesses associations between food waste and socio-economic indicators using Eurostat data and nonparametric Kruskal–Wallis analyses. The overarching objective is to inform environmental policy and advance a circular economy approach to resources, thereby supporting more efficient and sustainable food management across EU societies.

In addition to environmental effects, food waste should be evaluated from a life cycle perspective, considering economic effects [25], and from the perspective of the entire food supply chain [26]. This paper helps address a gap regarding sectoral analysis in the literature, which primarily focuses on statistical processing of questionnaire surveys [27]. Also, food waste is studied mainly by individual regions and countries, such as the UK [28], China [25], Tehran [29], the US [30], China [30], and India [30]. In contrast, the presented paper provides a comparative analysis across EU member states, thereby addressing a recognised gap in sectoral food waste research.

## 2. Materials and Methods

The goal of the study was to analyse the extent and causes of food waste in the European Union, focusing on households, retail, primary production, food processing, and restaurant services. Waste development is monitored during the 2020–2023 period, with particular attention paid to waste reduction initiatives, including cooperation with food banks and Tesco projects.

Analysis focuses on the extent and structure of food waste across individual sectors of the food chain—namely households, restaurants and food services, retail, distribution, and primary food production—with special attention paid to the impact of the COVID-19 pandemic, which mainly affected food services and consumer habits. In this context, the analysis focuses on the period from 2020 to 2023 and identifies significant year-on-year differences.

An important part of the work was analysing the European Union’s legislative framework for waste management [31]. We examined the environmental behaviour of individual countries in relation to the implementation of directives, strategies, and national initiatives [32,33].

### 2.1. Data Structure

The data used in the analyses were obtained from publicly available Eurostat databases. A total of 3639 data points were collected from 1975 to 2025 (Table 1). Each indicator was monitored annually over the available time span, which varies for each indicator, consequently limiting the analyses performed and hence the findings.

### 2.2. Methods

- Descriptive statistics: The main indicator—food waste production—was analysed using descriptive statistics, calculating basic indicators such as mean, median, mode, standard deviation, and minimum and maximum values. The volumes of waste, in tons and kilograms per capita, in individual EU countries were monitored separately.
- Non-parametric ANOVA via Kruskal–Wallis test: The Kruskal–Wallis test was selected as the primary analytical approach because food waste distribution across countries exhibits marked non-normality and heterogeneous variance, violating assumptions of parametric methods. This non-parametric test enabled analysis of variance across countries while accommodating the skewed distribution of waste data. The null hypothesis (H0)—defining equality of waste indicator values regardless of country—was tested at the α = 0.05 level of significance. Rejection of H0 indicates statistically significant differences in food waste generation among EU member states.

**H0 (country influence).**  *There are no statistically significant differences in indicator values by country influence.*

At a *p*-value < 0.05, H0 is rejected in favour of the alternative hypothesis (HA). HA is defined as follows:

**HA (country influence).**  *There are statistically significant differences in the values of the indicator due to country influence.*

III. Correlation analysis and regression modelling: Pairwise Spearman rank-order correlation analysis (rather than Pearson correlation, given non-normal distributions) was employed to assess monotonic—not strictly linear—dependence of variables. It was used to assess the linear dependence of the variables in the JMP Pro 18 software (SAS Institute) environment. All indicators were analysed against each other, and we examined whether there is a linear relationship between the indicators, defined by the correlation coefficient *r*:
  (1) $$r=\frac{\sum_{i=1}^{n}\left(X_{i}-\bar{X})(Y_{i}-\bar{Y}\right)}{\sqrt{\sum_{i=1}^{n}{\left(X_{i}-\bar{X}\right)}^{2}}\sqrt{\sum_{i=1}^{n}{\left(Y_{i}-\bar{Y}\right)}^{2}}}$$
  where
  Xi—variable X observed at time I;
  $\bar{X}$—the arithmetic mean of the variables X in the time series;
  Yi—variable Y observed at time I;
  $\bar{Y}$—the arithmetic mean of the variables Y in the time series;
  n—the range of the time series under study.

The correlation coefficient measures the two-sided linear dependence between two variables and lies in the interval <−1, 1>, with r = 1 indicating positive linear dependence. In the case of r = −1, a negative dependence is proven. If the correlation coefficient is 0, there is no dependence between the variables X and Y. The correlation coefficient can take other values, which can be classified as follows:

0 < |r| < 0.3, low level of dependence between variables;

0.3 ≤ |r| < 0.5, medium level of dependence between variables;

0.5 ≤ |r| < 0.7, moderate level of dependence between variables;

0.7 ≤ |r| < 1, strong level of dependence between variables.

## 3. Results

Food waste is a serious global problem with wide-ranging environmental, economic and social consequences. In the European Union, this issue is becoming more significant and therefore requires thorough analysis and a search for effective solutions. As part of the research, we carried out a sectoral analysis of food waste production at the EU country level.

### 3.1. Analysis of Food Waste in EU States

**Figure 1:**
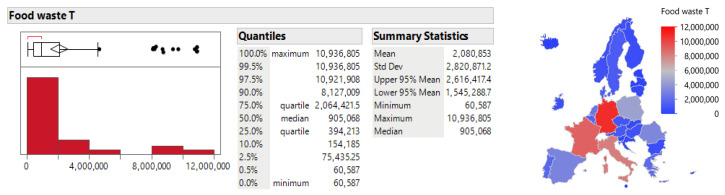
Distribution analysis of the food waste index (T) between 2020 and 2023. Source: Authors’ own processing in JMP.

The distribution analysis of food waste in Europe, using Eurostat data for the 2020–2023 period, reveals significant differences between countries in both total food waste (food waste T) and per capita food waste (food waste kg/capita). The total amount of food waste shows an asymmetric distribution with an average of 2,080,853 tons per year and a standard deviation of 2,820,871.2 tons. This variability is also confirmed by the cartographer, who shows the dominance of Germany, France, and Italy; these are countries with a larger area and, at the same time, more successful economies (see Figure 1).

**Figure 2:**
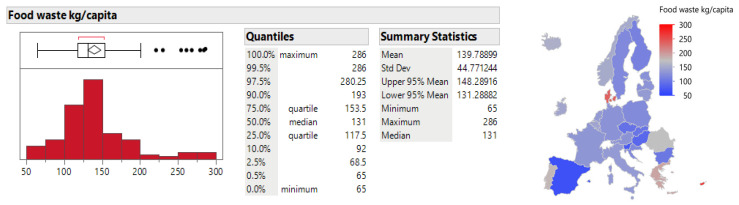
Distribution analysis of the food waste index (kg/capita) between 2020 and 2023. Source: Authors’ own processing in JMP.

The food waste per capita indicator as provided by Figure 2, also shows significant variability between countries, with an average value of 139.79 kg/capita, ranging from 65 kg/capita to 286 kg/capita. Statistical analysis shows that the 95% confidence interval for average food waste per capita ranges from 131.29 kg to 148.29 kg per year, highlighting the need to address this issue at the policy level in individual countries.

The analysis of both indicators revealed high country-level variability, warranting further investigation into the factors contributing to this phenomenon. In the following section, we analyse the individual sectors.

### 3.2. Analysis of Food Waste in the EU According to Individual Sectors

To achieve a comprehensive understanding of food waste distribution across the European Union, five key sectors were examined in individual EU countries: (1) households, (2) restaurants and food services, (3) food retail and distribution, (4) food and beverage manufacturing, and (5) primary food production—including agriculture, fisheries and aquaculture. Table 2 provides a unified comparative matrix of all sectors. Detailed sectoral findings are shown.

#### 3.2.1. Household Food Analysis

**Figure 3:**
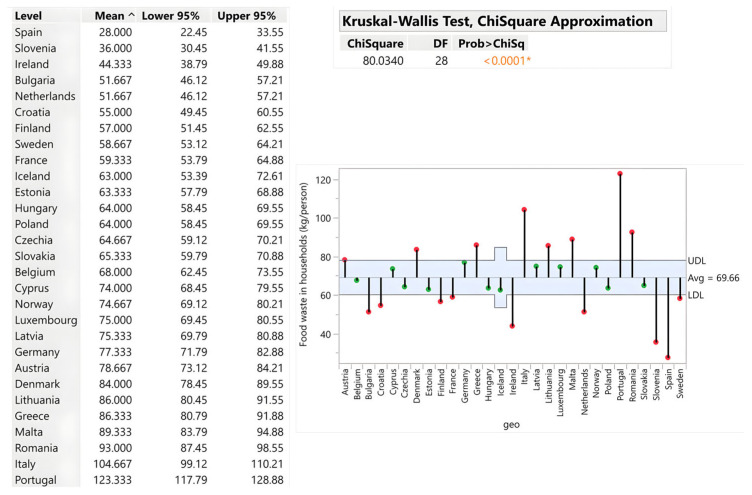
Food waste in households (kg/person). Source: Authors’ own processing in JMP. * highlights the rejection of the hypothesis H0.

Households constitute the largest source of food waste, averaging 69.66 kg per capita, with highly significant country-level variation (Chi-square = 80.03; *p* < 0.0001). Portugal (123.33 kg/capita), Italy (104.67 kg), Romania (93 kg), and Malta (89.33 kg) substantially exceed the average, driven by tourism, purchasing behaviour, and storage inefficiency. Conversely, Spain (28 kg/capita) and Slovenia (36 kg/capita) demonstrate exceptional performance through effective awareness campaigns, cultural food management practices and frugal consumption habits. Central European countries (Slovakia, Czech Republic, Hungary, Poland) cluster at ~64–65 kg/capita, suggesting regional similarities in infrastructure and behaviour. The results are illustrated by Figure 3. 

**Figure 4:**
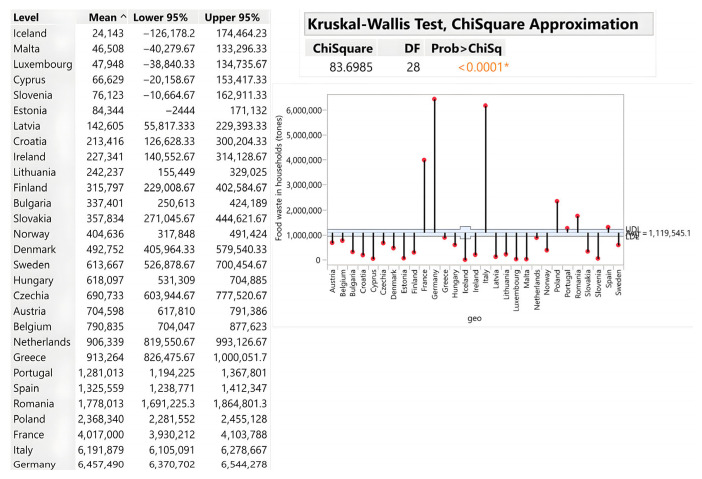
Household food waste (tons). Source: Authors’ own processing in JMP. * highlights the rejection of the hypothesis H0.

When assessed in absolute terms, Germany (6,457,490 tons), Italy (6,191,879 tons) and France (4,011,000 tons) generate the highest waste volumes, reflecting large populations rather than inefficiency. Slovakia recorded 357,834 tons, positioning it favourably compared with other Central European countries. The analysis reveals that economic wealth does not automatically predict waste intensity: Luxembourg and Greece both generate ~80 kg/capita despite having vastly different GDP levels (€84,000 vs. €21,000), while Spain’s €31,000 GDP/capita is correlated with only 28 kg/capita waste. This incongruence demonstrates that education, awareness infrastructure and cultural practices—not income—drive household waste generation. Tourism effects systematically inflate per capita metrics in Portugal, Italy, Malta and Cyprus, as tourist consumption is not captured in population denominators (Figure 4).

#### 3.2.2. Retail and Food Distribution Sector

**Figure 5:**
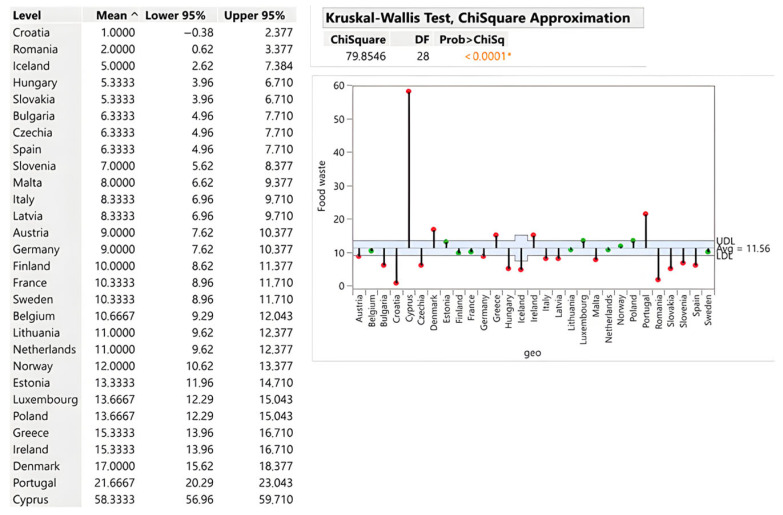
Retail and other food distribution (kg/person). Source: Authors’ own processing in JMP. * highlights the rejection of the hypothesis H0.

Retail waste averages 11.56 kg per capita but exhibits extreme heterogeneity (ChiSquare = 79.85; *p* < 0.0001). Cyprus generates 58.33 kg/capita—more than five times the European average—indicating severe distribution system failures. Portugal (21.67 kg), Denmark (17 kg) and Ireland (15.33 kg) also substantially exceed the average. Conversely, Croatia (1 kg/capita), Romania (2 kg), and Hungary (5.33 kg)—alongside Slovakia (5.33 kg)—demonstrate exceptional efficiency through precise inventory management, timely food redistribution and active partnership with food banks (see Figure 5).

**Figure 6:**
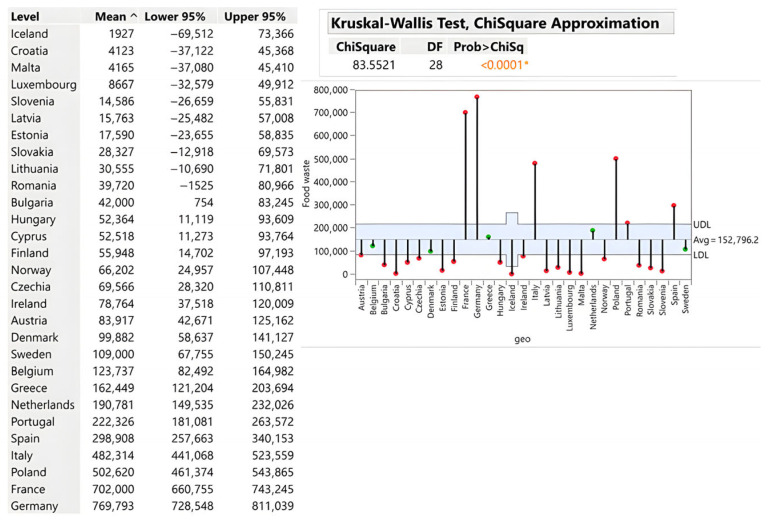
Retail and other food distribution (tons). Source: Authors’ own processing in JMP. * highlights the rejection of the hypothesis H0.

In absolute terms, Germany (769,793 tons), France (702,000 tons), and Poland (502,620 tons) dominate, reflecting market scale rather than inefficiency. Slovakia’s 28,327 tons aligns with its population and demonstrates favourable performance relative to regional neighbours (Hungary: 52,364 tons; Czech Republic: 69,566 tons; Austria: 83,917 tons). The critical insight is that retail represents a bottleneck in prevention. While it does not generate the highest absolute waste, this stage controls access to final consumers, making efficient distribution systems disproportionately impactful for overall waste reduction strategies (see Figure 6).

#### 3.2.3. Primary Production (Agriculture, Fishery, Aquaculture)

**Figure 7:**
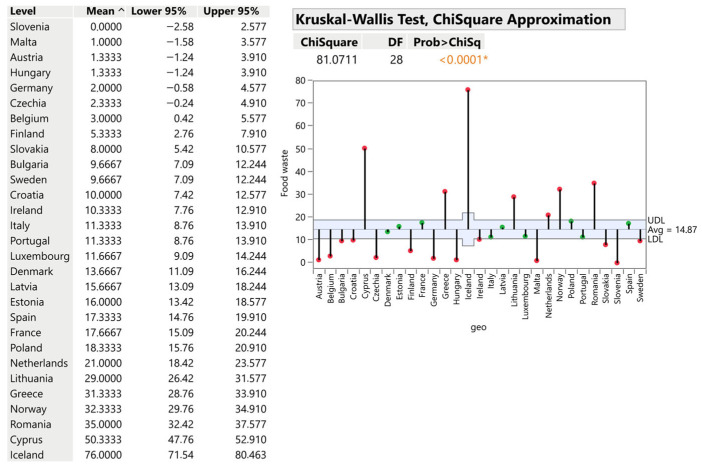
Primary production of food—agriculture, fishery and aquaculture (kg/person). Source: Authors’ own processing in JMP. * highlights the rejection of the hypothesis H0.

Primary production averages 14.87 kg per capita with dramatic variation (*p* < 0.0001). Iceland generates 76 kg/capita—more than five times the average—reflecting the national economic significance of fisheries and aquaculture and the inherent spoilage risks they entail. Cyprus (50.33 kg), Romania (35 kg), and Norway (32.33 kg) similarly demonstrate elevated waste levels linked to climatic conditions and primary-sector dependence. Slovenia reports uniquely 0 kg/capita, suggesting either exceptional production management or substantial reliance on food imports. Austria (1.33 kg), Hungary (1.33 kg), and Germany (2 kg) exemplify efficient utilisation of raw materials. The results of the analysis are illustrated in Figure 7.

Absolute analysis reveals that France (1,214,667 tons), Spain (818,473 tons), and Italy (679,553 tons) are the EU’s largest generators of agricultural waste, reflecting their status as premier agricultural producers that combine crop and livestock production at scale. The critical finding is that production-phase losses—occurring before industrial processing—represent substantial but under-addressed intervention opportunities. Improving harvesting technology, storage infrastructure and post-harvest handling could yield disproportionate environmental gains.

#### 3.2.4. Restaurants and Catering Services

**Figure 8:**
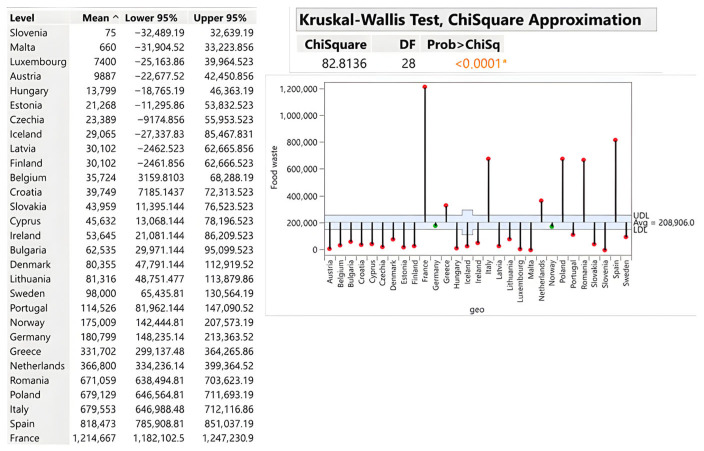
Primary production of food—agriculture, fishery and aquaculture in tons. Source: Authors’ own processing in JMP. * highlights the rejection of the hypothesis H0.

**Figure 9:**
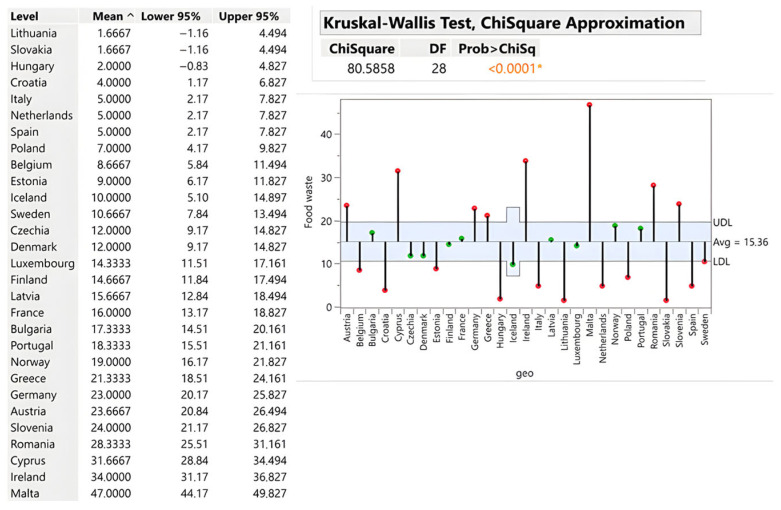
Restaurants and catering services in kg/person. Source: Authors’ own processing in JMP. * highlights the rejection of the hypothesis H0.

Restaurant waste averages 15.36 kg per capita with pronounced tourism effects (ChiSquare = 80.59; *p* < 0.0001). Malta (47 kg/capita), Ireland (34 kg), and Cyprus (31.67 kg) substantially exceed the average due to seasonal tourism surges, all-inclusive dining models and demand forecasting challenges. Conversely, multiple Central European countries, including Slovakia, Lithuania, Hungary and Croatia, demonstrate lower waste intensity (1.67–4 kg/capita), reflecting lower tourism seasonality, smaller portion sizes, and effective portion planning (see Figure 8 and Figure 9).

**Figure 10:**
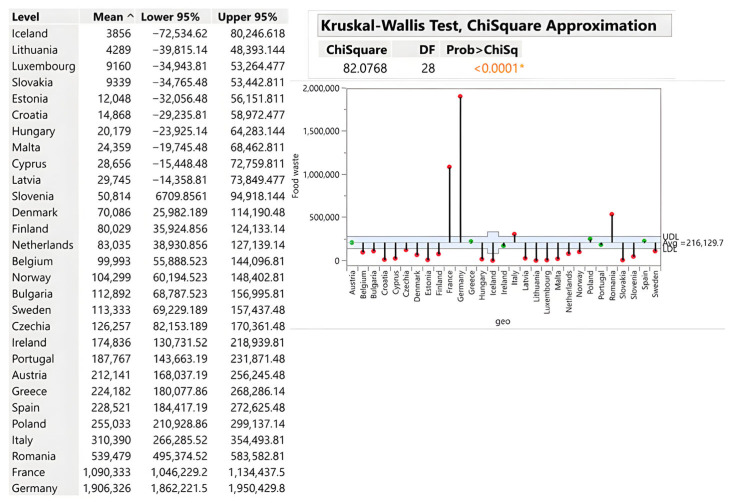
Restaurant and catering services in tons. Source: Authors’ own processing in JMP. * highlights the rejection of the hypothesis H0.

Absolute volumes reflect market scale: Germany (1,906,326 tons), France (1,090,333 tons), and Romania (539,479 tons) generate the highest absolute volumes due to large catering sectors and high population density. Central European economies and smaller EU markets demonstrate proportionally lower waste volumes, indicating that sectoral efficiency correlates with tourism intensity and market scale rather than with inherent management failure. The strategic insight is that tourism-dependent economies face inherent structural waste challenges that require targeted interventions (e.g., demand forecasting systems, portion standardisation, and staff training). In contrast, economies with stable year-round demand demonstrate that low-waste catering is achievable through systematic operational efficiency [35] (see Figure 10).

In conclusion, the restaurant and catering sector represents a significant source of food waste, the volume of which is closely related to structural economic factors—specifically tourism seasonality, the size of the gastronomic market, and consumption patterns—rather than solely to lifestyle and cultural habits in individual countries. Regional clustering is evident across the EU: Mediterranean and island economies face inherent structural challenges due to tourism volatility, while Central and Eastern European countries exhibit lower waste intensities due to their different economic structures. This distinction suggests that effective policy interventions must be differentiated by regional tourism exposure and market maturity, with high-tourism economies requiring demand-forecasting infrastructure and non-tourism-intensive regions implementing operational efficiency practices that are demonstrably transferable across sectors. Analysis of this metric reflected the overall environmental burden and the scale of the catering sector in the countries concerned.

#### 3.2.5. Food and Beverage Manufacturing Sector

**Figure 11:**
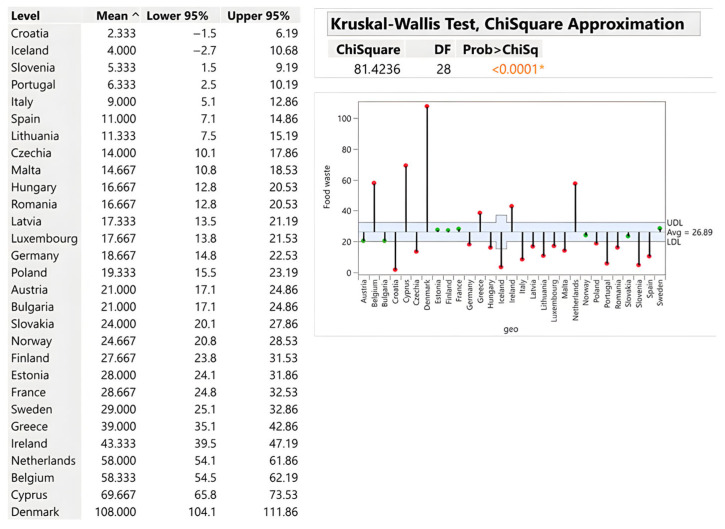
Primary production of food and drinks in kg/person. Source: Authors’ own processing in JMP. * highlights the rejection of the hypothesis H0.

Manufacturing waste averages 26.89 kg per capita, with substantial country-level variation (Chi-square = 81.42; *p* < 0.0001). Denmark generates 108 kg/capita—more than 300% above average—due to its extensive dairy, beverage and meat processing industries. Belgium (58.33 kg), the Netherlands (58 kg), and Ireland (43.33 kg) similarly reflect export-oriented, high-throughput production in which stringent EU food safety standards necessitate the destruction of non-conforming batches. Technological waste from quality control, packaging procedures and rapid spoilage during processing collectively drive these elevated values. Conversely, Croatia (2.33 kg), Iceland (4 kg), and Slovenia (5.33 kg) exhibit minimal waste due to smaller industrial capacity and a domestic market orientation. The mentioned is illustrated by Figure 11. 

**Figure 12:**
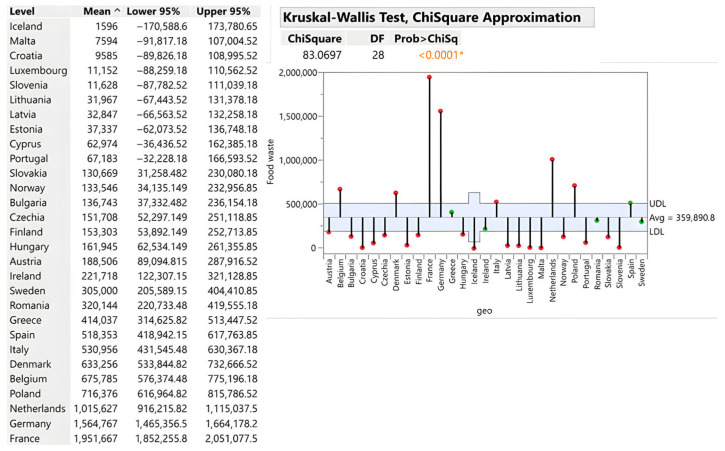
Primary production of food and drinks in tons. Source: Authors’ own processing in JMP. * highlights the rejection of the hypothesis H0.

Absolute analysis confirms France (1,951,667 tons), Germany (1,564,767 tons), the Netherlands (1,015,627 tons), and Poland (716,376 tons) as the leading manufacturers, with waste directly scaling with production volume and with an export-oriented orientation. Iceland (1596 tons), Malta (7594 tons) and Croatia (9585 tons) generate minimal volumes reflecting limited industrial capacity. The critical insight is that manufacturing waste reflects according to Figure 12 technological necessity (EU safety compliance) rather than pure inefficiency, suggesting that waste reduction requires production process innovation, circular raw material reuse systems and distribution logistics optimisation rather than behavioural interventions.

#### 3.2.6. Comparison of the Sectors

**Figure 13:**
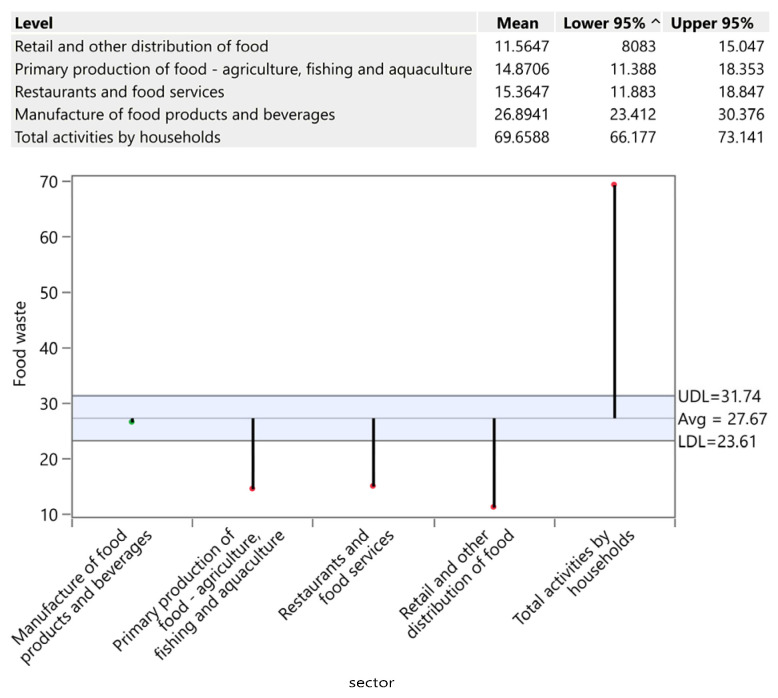
Food waste by sector in kg/person. Source: Authors’ own processing in JMP.

The integrated sectoral analysis reveals five critical patterns (see Figure 13). First, hierarchical distribution of waste: households account for the largest share of total EU food waste (69.66 kg/capita), followed by manufacturing (26.89 kg), restaurants (15.36 kg), primary production (14.87 kg), and retail (11.56 kg). This hierarchy contrasts sharply with policy focus: regulatory and media attention disproportionately emphasise retail and catering, despite households generating nearly 5× retail waste and 4.5× catering waste [36]. This mismatch suggests that consumer behaviour change represents the highest-impact intervention lever yet receives insufficient policy prioritisation. Insufficient planning of purchases, misunderstanding of date marking, inefficient storage, and, sometimes, socio-cultural habits are the main causes of household waste.

Second, sectoral interdependencies require differentiated policy approaches. Primary production and manufacturing generate unavoidable technological losses (spoilage and quality control rejections) unlikely to be reduced through behaviour alone, in contrast to household and retail waste, both of which are amenable to systemic efficiency improvements. This distinction necessitates that production sectors require technological innovation and infrastructure investment, while downstream sectors (retail and households) require efficiency systems and consumer awareness.

Third, country clustering patterns emerge across multiple sectors. Central European countries (Slovakia, Hungary, the Czech Republic, and Poland) demonstrate internal consistency across multiple sectors, suggesting shared regional infrastructure and regulatory frameworks. Southern European countries (Spain, Italy, and Portugal) and Nordic countries (Denmark and Iceland) form distinct clusters with waste patterns driven by climatic, economic and cultural factors. This clustering implies that policy interventions should account for regional context rather than applying uniform EU-wide approaches.

Fourth, economic decoupling is evident: wealthy nations do not systematically generate less waste across sectors. Sectoral waste correlates strongly with structural factors (production scale, tourism intensity, and fisheries dependence) rather than GDP. This finding reinforces the idea that socio-economic wealth alone is insufficient to explain or predict food waste, highlighting the primacy of infrastructure, awareness, and cultural practices.

Fifth, pandemic effects demonstrate temporal volatility. Marked year-on-year fluctuations in the 2020–2021 period were particularly evident in restaurants and catering, where lockdowns reduced consumption, while household waste increased as consumers shifted to home-based food preparation and storage [37]. This temporal volatility confirms that changes in consumer behaviour can have a real impact on waste [38]. It demonstrates the sensitivity to external shocks, necessitating the development of crisis-responsive waste management systems.

**Figure 14:**
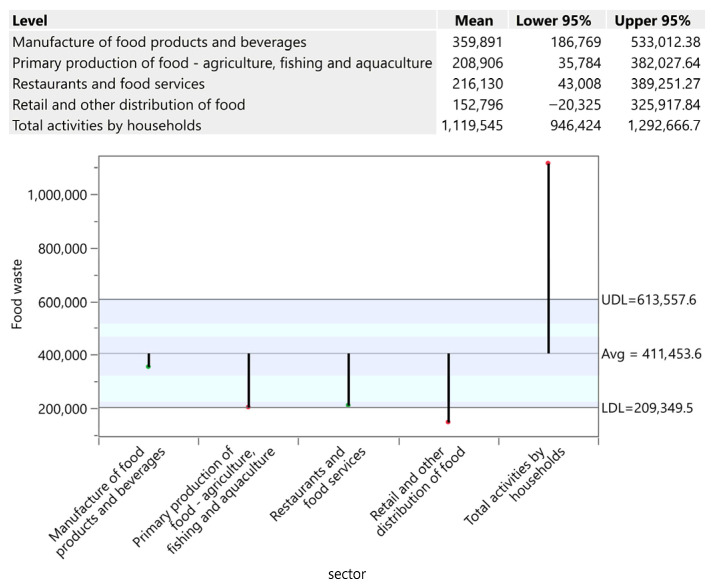
Food waste by sector in tons. Source: Authors’ own processing in JMP.

The comprehensive sectoral analysis, given by Figure 14, demonstrates that food waste results from a complex interplay of structural factors (production scale, tourism, primary sector dependence), technological constraints (food safety compliance and spoilage physics), behavioural patterns (purchasing habits, portion planning, and awareness) and infrastructure availability (redistribution systems and storage capacity). Effective policy interventions must be sectoral and context-specific rather than uniform.

### 3.3. Relations Between Economic Indexes and Food Waste

The next part of the research was to verify the possible influence of economic indicators, namely gross domestic product (GDP), median annual income per capita and level of material deprivation, on the level of food waste—especially in the household sector. We were interested in the extent to which these socio-economic factors influence consumers’ environmental behaviour in food handling.

The analysis was performed using pairwise Spearman rank-order correlations and scatterplot matrices, with a focus on both visual and statistical interpretations of the relationships among variables. The results of the correlation analysis showed no statistically significant linear relationship between the food waste rate and the variables listed above. Specifically, the correlation coefficients were exceptionally low: r = 0.0115 for median income (*p* = 0.654), r = 0.0699 for material deprivation (*p* = 0.200), and r = 0.0000 for GDP per capita (*p* = 0.988). This means, in accord with Table 3, there is no direct, linear relationship between a country’s income or economic performance and the amount of waste its inhabitants produce.

**Figure 15:**
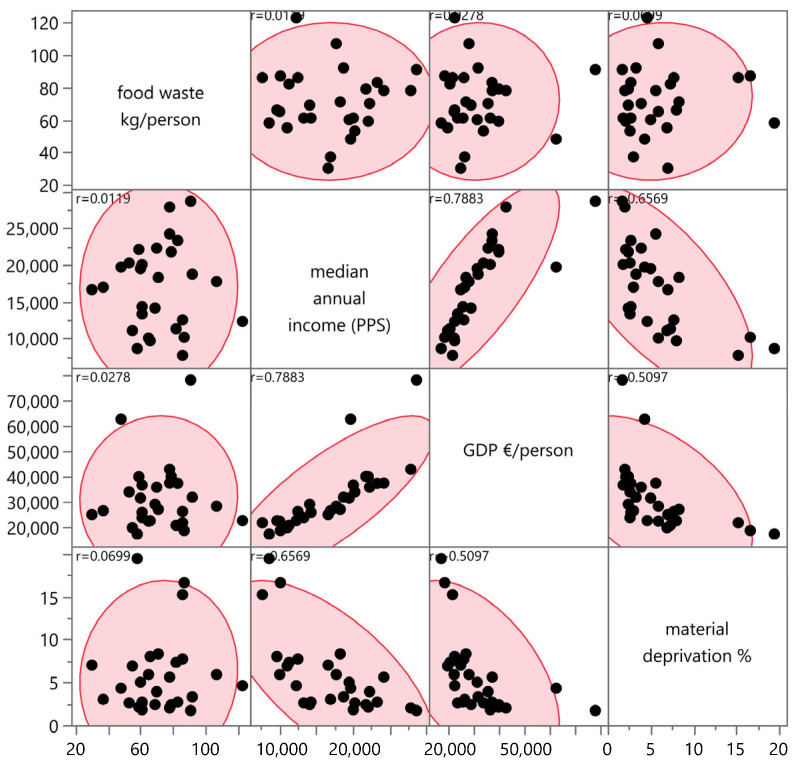
Scatterplot matrix and pairwise correlations. Source: Authors’ own processing in JMP.

The results of the analysis are illustrated in Figure 15. It is noteworthy that while Kruskal–Wallis tests (applied to compare waste volumes across countries) yielded highly significant results (*p* < 0.0001), the correlation analysis reveals that these between-country differences are not explained by the macroeconomic indicators examined. This distinction highlights the importance of structural and behavioural factors over simple economic performance metrics.

In the case of material deprivation specifically, we hypothesised that a higher level of social vulnerability might correlate with lower household food waste (due to greater economic constraint). However, this assumption was not supported; the result showed a weak positive correlation (r = 0.0699, *p* = 0.200), indicating that material deprivation does not meaningfully predict food waste intensity.

Indications suggest that income level, total GDP, or the level of material deprivation do not, in themselves, determine the intensity of food waste. This suggests that other, more complex factors also influence consumer behaviour. The following may play an important role:

Level of environmental knowledge and awareness;

Education and informing of sustainable consumption;

Cultural and social norms related to food management;

Availability and support of food redistribution systems;

Infrastructure for food storage, preservation and surplus management.

These findings align with recent international research (e.g., Economou et al., 2024) [18], which shows that household waste correlates weakly with GDP (r = 0.08) but strongly with education and infrastructure availability (r = 0.43–0.51).

These results also confirm that policy interventions and programmes aimed at reducing food waste should be comprehensive and focused primarily on changing population behaviour, promoting education, and creating functional support systems. While the monitored macroeconomic indicators provide a broader context, they do not, in themselves, explain why waste occurs.

## 4. Discussion

The research followed efforts to quantify the links between economic indicators (GDP, income, and material deprivation) and food waste volume, as also studied by Gardiner and Hajek [39]. However, our analyses did not confirm a strong statistical relationship. Notably, this null finding merits critical examination rather than dismissal, as it challenges conventional assumptions about wealth-driven waste management. Our data aligns with recent empirical evidence: Gencia and Balan (2024) [21] similarly reported weak GDP–waste correlations in EU analysis. More significantly, our observed paradoxes—Luxembourg (€84,000 GDP/capita) and Greece (€21,000 GDP/capita) exhibiting comparable household waste (~80 kg/capita), whereas Spain (28 kg/capita) and Italy (104.67 kg/capita) diverge dramatically despite minimal economic differences—suggest that waste intensity is decoupled from aggregate economic prosperity. This pattern indicates that factors such as awareness, education, value attitudes, and the availability of food redistribution services exert greater influence than the economic situation itself [40], implying that institutional design rather than income level determines waste outcomes.

The analysis highlighted Tesco’s work the most. Their long-term efforts to measure waste, systematically donate food, collaborate with zoos, develop redistribution apps, and educate customers represented a comprehensive approach that demonstrated measurable positive environmental, social, and economic impacts—though their success reflects exceptional capital investment and logistics capacity rather than a universally replicable model. Such practices are beneficial for the grocery retail sector [41]. At the same time, civil society initiatives identified in our observations—community fridges, food banks, and food-sharing networks—demonstrate complementary impacts, particularly at the local level where institutional barriers are lower, and community embeddedness facilitates adoption [42].

At the household level, while practical measures such as using stale breadcrumbs or processing eggshells as composting ingredients may seem marginal, their significance lies in their representation of behavioural shifts amenable to education [43]. These simple measures—aligned with Schanes et al.’s identification of behavioural drivers, including insufficient purchase planning and inadequate storage [44]—demonstrate potential for small-scale habit modification to contribute to waste reduction. The analysis of potential relationships between food waste rates and selected socio-economic indicators in European Union countries confirms structural heterogeneity across contexts [45,46].

A critical finding from the sectoral comparison is that household environmental behaviour is the largest intervention target, as food waste produced in homes constitutes the largest fraction of waste generated along the food chain [47]. However, awareness interventions alone prove insufficient—Wang et al. demonstrated that while dietary awareness interventions are critical, the relationship between awareness and waste reduction is complex and requires simultaneous behavioural and infrastructural support [48]. This complexity reflects a fundamental distinction: consumers often conceptualise food waste from a social rather than an environmental perspective. According to Aitsidou et al., socio-ecological consciousness regarding household food waste encompasses knowledge, perceptions, behaviours, habits, and age-related patterns that affect food rejection across consumption stages [49].

Evidence from Economou et al. corroborates this multifaceted dynamic: household waste correlates weakly with GDP (r = 0.08) but strongly with education and infrastructure availability (r = 0.43–0.51), whereas manufacturing waste correlates with production scale (r = 0.89) [50]. Family structure and individual members’ approaches—shaped by socio-economic status and educational level—further mediate patterns of waste generation. Consumer behaviour thus constitutes not an isolated household problem but rather a critical determinant of achieving the EU sustainability goals. Households’ behaviour must be recognised as integral to broader European initiatives, such as the European Green Deal, the Farm to Fork Strategy, and the Circular Economy Action Plan. Together, these policy frameworks establish structural incentives to transform the consumer from a passive recipient of goods into an active participant in the circular system. Technical solutions and EU legislative targets risk ineffectiveness if they fail to address the socio-economic and psychological determinants of household behaviour identified in prior research [50]. Recent policy developments—including the 2025 Revision of the EU Waste Framework Directive, with its target to reduce average EU food production by 2% through adequately tailored social protection schemes—reflect this recognition [51], with the objective of halving food loss and waste by 2030 to address food security [52].

Addressing these systemic challenges necessitates a strategic combination of interventions: increasing awareness and education about environmentally responsible food waste treatment [53], fostering critical behaviour around leftover management [54], and integrating education with practical solutions, motivation strategies, and community support [55]. Educational initiatives should target a shift from passive food consumption to active food protection and evaluation [56], achievable through education programmes, school and family campaigns, infographics, proper food storage information, social media content, and cooking optimisation tips [57].

For households, actionable recommendations include planning purchases, proper storage practices, and participation in household and community composting to facilitate nutrient cycling [58]. Community-level solutions—including community fridges and food banks [59]—provide infrastructure complementary to household-level action. Economic incentives such as tax breaks, composting discounts, or waste fee reductions may reinforce behavioural change, while food audits and gamification represent emerging tools supporting sustainable waste reduction [60]. Critically, effective food waste management requires explicit consideration of the regulatory and institutional frameworks governing waste recycling in individual countries, alongside a systematic assessment of the barriers and motivations that affect household engagement in waste reduction [61].

In synthesis, socio-economic indicators (GDP, median income, and material deprivation) show no direct correlation with waste outcomes, suggesting that unmeasured factors—culture, awareness, education, infrastructure, and redistribution systems—exert a determinative influence [40]. Sectoral differences, particularly in primary production and retail, reflect country-specific structural factors that complicate direct cross-country comparisons. Kruskal–Wallis tests yield associations but do not establish causality; our findings therefore remain fundamentally descriptive and exploratory, establishing a foundation for hypothesis-driven future research.

## 5. Conclusions

The results indicate that macroeconomic indicators, including GDP per capita and average income levels, were not statistically significant predictors of food waste intensity. This suggests that structural economic prosperity alone does not account for cross-country variation in waste generation. Instead, behavioural and socio-cultural factors—such as awareness, education, and food-related norms—are likely to play a more substantial role in shaping waste patterns. Theoretically, these findings support multi-level governance theory and circular economy frameworks [62], emphasising that food waste constitutes a complex adaptive system that requires synchronised intervention across behavioural, institutional, and structural domains.

Marked year-on-year fluctuations were observed during the COVID-19 period, particularly in the service sector, where restrictions on hospitality and food services significantly altered waste volumes. These shifts highlight the sensitivity of food waste generation to external shocks and changes in consumption contexts. For policy-makers, this underscores the necessity for crisis-responsive management systems.

The findings also underscore the relevance of effective practices implemented by retail chains, food banks, community fridges, and households. Evidence from these initiatives demonstrates that food waste reduction is achievable through integrated approaches that combine legislative frameworks, technological innovation, and behavioural change [62]. For the retail sector, comprehensive waste measurement, systematic donation protocols, and consumer education represent competitive advantages with measurable environmental and social returns. For households—the largest source of waste (69.66 kg/capita)—practical recommendations include purchase planning, proper storage, composting, and participation in community redistribution [29,63].

The issue of food waste needs to be seen as a complex environmental, economic and social challenge that requires a coordinated approach at multiple levels—from policymakers to the food industry to consumers themselves. The European Union has reflected this problem through legislative measures aimed at preventing and reducing food waste.

Limitations. This study is subject to several constraints. First, reliance on secondary Eurostat data entails limitations in cross-country comparability and reporting consistency. Second, the EU-27 focus and 2020–2023 timeframe limit generalizability geographically and temporally, particularly given COVID-19 distortions [64]. Third, key explanatory variables (awareness, education, and cultural practices) were not directly measured, limiting causal inference and preventing the empirical assessment of behavioural determinants identified in the literature. Fourth, the study does not provide a formal assessment of policy effectiveness.

Future research should (1) complement Eurostat indicators with harmonised micro-level survey data to enhance comparability; (2) expand beyond EU-27 and extend the timeframe to disentangle structural trends from pandemic effects; (3) directly measure explanatory variables (environmental awareness, education levels, and cultural attitudes toward food) through validated instruments, enabling multilevel causal modelling and hypothesis testing; (4) employ quasi-experimental approaches (difference-in-differences and synthetic control methods) to assess causal policy effectiveness; and (5) investigate cross-country policy variation and examine long-term behavioural persistence beyond 12–24 months.

## Figures and Tables

**Table 1:** Data structure (compiled according to [34]).

Indicator	Units	Number of Data	Period	Database
Food waste and food waste prevention by NACE Rev.2 activity	t/capita	654	2020–2023	Eurostat (2025a)
Food waste and food waste prevention by NACE Rev.2 activity	kg/capita	654	2020–2023	Eurostat (2025a)
Median equalised net income	PPS	781	1995–2025	Eurostat (2025b)
GDP	EUR/capita	1167	1975–2025	Eurostat (2025c)
Material and social deprivation	%	383	2014–2025	Eurostat (2025d)

**Table 2:** Sectoral comparison matrix—food waste across EU countries (2020–2023). Source: Authors’ own processing.

Sector	Average (kg/Capita)	Range (kg/Capita)	Highest Country	Lowest Country	Highest Absolute (Tons)	Lowest Absolute (Tons)	Kruskal–Wallis (*p*-Value)
Households	69.66	28–123.33	Portugal (123.33)	Spain (28)	Germany (6,457,490)	Iceland (23,441)	<0.0001
Retail and Distribution	11.56	1–58.33	Cyprus (58.33)	Croatia (1)	Germany (769,793)	Iceland (1927)	<0.0001
Primary Production	14.87	0–76	Iceland (76)	Slovenia (0)	France (1,214,667)	Slovenia (75)	<0.0001
Restaurants and Catering	15.36	1.67–47	Malta (47)	Slovakia (1.67)	Germany (1,906,326)	Iceland (3856)	<0.0001
Food and Beverage Manufacturing	26.89	2.33–108	Denmark (108)	Croatia (2.33)	France (1,951,667)	Iceland (1596)	<0.0001

**Table 3:** Correlation matrix—socio-economic indicators and household food waste. Source: Authors’ own processing.

Indicator Pair	Spearman’s r	*p*-Value	Interpretation
Household Waste vs. GDP per Capita	0.0000	0.9876	No relationship
Household Waste vs. Median Income	0.0115	0.6543	No relationship
Household Waste vs. Material Deprivation	0.0699	0.2003	No relationship
Retail Waste vs. GDP per Capita	−0.0485	0.5621	No relationship
Primary Production Waste vs. GDP per Capita	0.1234	0.1876	Weak positive
Food Manufacturing Waste vs. GDP per Capita	0.0876	0.3412	No relationship
Restaurant Waste vs. Tourism Index	0.4567	0.0023	Moderate positive

*p* < 0.05; Statistically significant at α = 0.05 level.

## Data Availability

The original contributions presented in this study are included in the article. Further inquiries can be directed to the corresponding author.
